# Thermodynamic Model for B-Z Transition of DNA Induced by Z-DNA Binding Proteins

**DOI:** 10.3390/molecules23112748

**Published:** 2018-10-24

**Authors:** Ae-Ree Lee, Na-Hyun Kim, Yeo-Jin Seo, Seo-Ree Choi, Joon-Hwa Lee

**Affiliations:** Department of Chemistry and RINS, Gyeongsang National University, Gyeongnam 52828, Korea; dldofl24@naver.com (A.-R.L.); nahoney31@gmail.com (N.-H.K.); darkfntlvj@naver.com (Y.-J.S.); csr2915@nate.com (S.-R.C.)

**Keywords:** Z-DNA, DNA-protein interaction, B-Z transition, Z-DNA binding protein

## Abstract

Z-DNA is stabilized by various Z-DNA binding proteins (ZBPs) that play important roles in RNA editing, innate immune response, and viral infection. In this review, the structural and dynamics of various ZBPs complexed with Z-DNA are summarized to better understand the mechanisms by which ZBPs selectively recognize d(CG)-repeat DNA sequences in genomic DNA and efficiently convert them to left-handed Z-DNA to achieve their biological function. The intermolecular interaction of ZBPs with Z-DNA strands is mediated through a single continuous recognition surface which consists of an α3 helix and a β-hairpin. In the ZBP-Z-DNA complexes, three identical, conserved residues (N173, Y177, and W195 in the Zα domain of human ADAR1) play central roles in the interaction with Z-DNA. ZBPs convert a 6-base DNA pair to a Z-form helix via the B-Z transition mechanism in which the ZBP first binds to B-DNA and then shifts the equilibrium from B-DNA to Z-DNA, a conformation that is then selectively stabilized by the additional binding of a second ZBP molecule. During B-Z transition, ZBPs selectively recognize the alternating d(CG)*_n_* sequence and convert it to a Z-form helix in long genomic DNA through multiple sequence discrimination steps. In addition, the intermediate complex formed by ZBPs and B-DNA, which is modulated by varying conditions, determines the degree of B-Z transition.

## 1. Introduction

Left-handed Z-DNA is a higher energy conformation than right-handed B-DNA. Z-DNA was first found in a polymer of alternating d(CG)*_n_* DNA duplexes observed in high salt conditions [1]; its crystal structure was reported in 1979 [2]. The Z-DNA helix is built from d(CG)-repeats, with the dC in the *anti*-conformation and the dG in the unusual *syn*-conformation, which causes the backbone to follow a zigzag path [3,4]. Z-DNA can also be stabilized by negative supercoiling generated behind a moving RNA polymerase during transcription [5].

**Figure 1:**
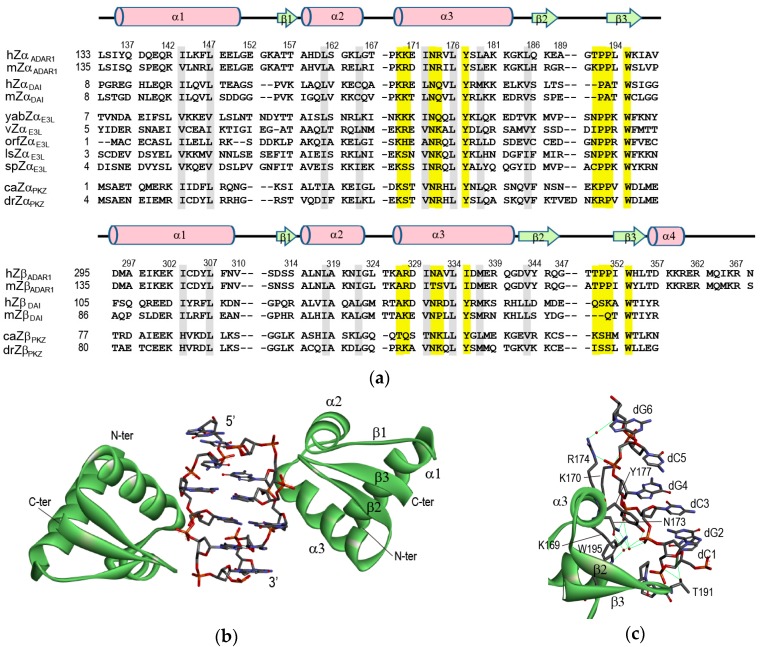
(**a**) Multiple sequence alignment of ZBPs: hZα_ADAR1_, hZβ_ADAR1_, human ADAR1; mZα_ADAR1_, mZβ_ADAR1_, murine ADAR1; hZα_DAI_, hZβ_DAI_, human DAI; mZα_DAI_, mZβ_DAI_, murine DAI; yabZα_E3L_, Yaba-like disease virus E3L; vZα_E3L_, vaccinia virus E3L; orfZα_E3L_, orf virus E3L; lsZα_E3L_, lumpy skin disease virus E3L; spZα_E3L_, swinepox virus E3L; caZα_PKZ_, caZβ_PKZ_, goldfish PKZ; drZα_PKZ_, drZβ_PKZ_, zebrafish PKZ. Numbering and secondary structural elements for hZα_ADAR1_ and hZβ_ADAR1_ are shown above the sequence. Yellow and gray bars indicate residues important for Z-DNA recognition and protein folding, respectively. (**b**) Overview of the hZα_ADAR1_ domain bound to left-handed Z-DNA (PDB id: 1QBJ) [7]. (**c**) View of the DNA recognition surface of hZα_ADAR1_ (PDB id: 1QBJ) [7]. The green lines indicate the H-bonding interactions. In (**b**,**c**), the backbone structure of hZα_ADAR1_ domain and Z-DNA duplex, d(TCGCGCG)_2_, are represented by the green ribbon and element-based stick presentation, respectively.

A distinct biological function of Z-DNA is suggested by the discovery of various Z-DNA binding proteins (ZBPs). Double stranded (ds) RNA deaminase 1 (ADAR1) deaminates adenine in pre-mRNA to yield inosine, which codes as guanine [6,7,8]. ADAR1 has two left-handed Z-DNA binding domains (ZBDs), Zα and Zβ, at its NH_2_-terminus [7,9]. High binding affinity of this Zα domain to Z-DNA was shown by a band-shift assay and confirmed by CD and Raman spectroscopic measurement [10,11,12]. The DNA-dependent activator of IFN-regulatory factors (DAI; also known as ZBP1 or DLM-1) also contains two tandem ZBDs (Zα and Zβ) at the NH_2_-terminus, such as ADAR1 [13,14]. It has been shown that ZBDs regulate the localization of DAI and its association with stress granules [15,16]. All poxviruses have a gene called E3L that consists of two domains: An N-terminal ZBD and a C-terminal RNA binding domain [17,18]. This ZBD shows sequence homology to the Zα domains found in human ADAR1 and in the DAI of mammals (Figure 1a). The Z-DNA binding affinity of E3L protein is essential for pathogenesis in the poxviruses [17,18,19]. The RNA-dependent protein kinase (PKR) plays an important role in the innate immune response against viral infections by recognizing dsRNA in the cytosol [20,21,22]. In fish species, a functional analogue of PKR, PKZ contains two ZBDs instead of dsRNA binding domains [23,24,25,26]. Similar to PKR, the phosphorylation function of PKZ is activated by Z-DNA binding [24].

These biological data, along with the results of more recent structural studies of Z-DNA induced by various ZBPs, have provided insights into Z-DNA recognition and ZBP-induced B-Z transition carried out by the innate immune response, viral infection, and RNA editing that are influenced by the nature of the Z-DNA. Crystallographic and NMR studies have provided detailed three-dimensional (3D) structures and dynamic information of various ZBPs complexed with Z-DNA. The structural and dynamic data summarized in this review have yielded a rich understanding of the mechanisms by which ZBPs selectively recognize the d(CG)-repeat DNA sequences in genomic DNA and efficiently convert them to left handed Z-DNA to achieve their biological function.

## 2. Crystal Structures of ZBPs Complexed with DNA Duplexes

### 2.1. hZα_ADAR1_-Z-DNA Complex

In 1999, Alexander Rich and his colleagues first reported the crystal structure of the Zα domain of human ADAR1 (hZα_ADAR1_) complexed with a six-base-pair (bp), double-stranded (ds) DNA fragment d(TCGCGCG)_2_ [7]. The monomeric hZα_ADAR1_ domain binds to one strand of the palindromic dsDNA, in which the conformation of the DNA substrate is very similar to the canonical Z-DNA structure. (Figure 1b) [7]. A second monomer binds to the opposite strand of DNA yielding two-fold symmetry with respect to the DNA helical axis (Figure 1b) [7]. The hZα_ADAR1_ domain has a compact α/β architecture containing a three-helix bundle (α1 to α3) and twisted antiparallel β sheets (β1 to β3) (Figure 1b). The arrangement of a β-hairpin with hydrogen-bonds (H-bonds) between the β2 (L185–A188) and β3 strands (P193–I197) is a common feature of helix-turn-helix (HTH) proteins with a/β topology [7]. Aliphatic residues from the three helices, together with the W195 in strand β3, form a hydrophobic core [7]. The NMR studies reported that the free hZα_ADAR1_ protein in the solution adopts a similar fold in its complex structure [9,27].

**Figure 2:**
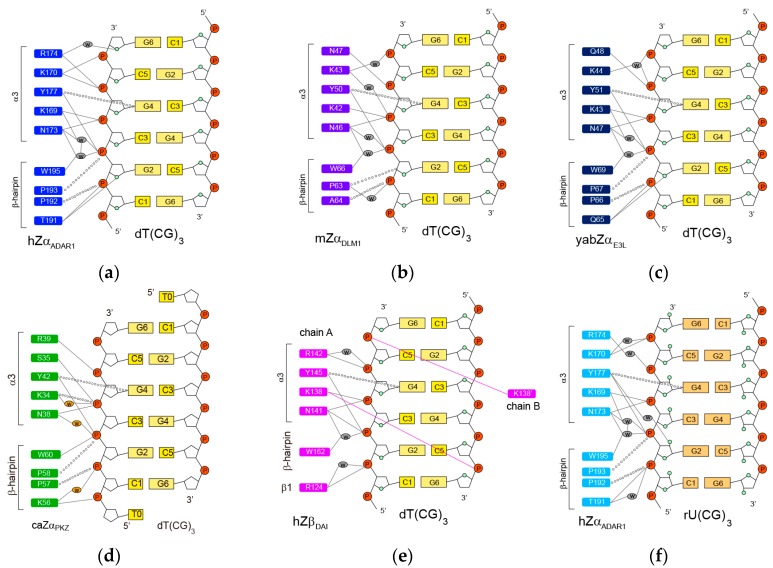
Protein residues involved Z-DNA/Z-RNA interactions in (**a**) hZα_ADAR1_–dT(CG)_3_ [7], (**b**) mZα_DLM1_–dT(CG)_3_ [29], (**c**) yabZα_E3L_–dT(CG)_3_ [30], (**d**) caZα_PKZ_–dT(CG)_3_ [31], (**e**) hZβ_DAI_–dT(CG)_3_ [32], and (**f**) hZα_ADAR1_–rU(CG)_3_ complexes [33]. Intermolecular H-bonds and van der Waals contacts are indicated by solid lines and open circles, respectively. The water molecules in key positions within the protein–DNA interface are indicated by ovals.

The contact of hZα_ADAR1_ with the Z-DNA strand is mediated through a single continuous recognition surface, which consists of residues from an α3 helix and a β-hairpin (β2-loop-β3, also called a β-wing) (Figure 1c) [7]. The electrostatic interactions in the complex are made between K169, K170, N173, R174, and Y177 in an α3 helix as well as between T191 and W195 in a β-hairpin and five consecutive phosphate-backbones of Z-DNA (Figure 1c) [7]. K169 and N173 form the direct and water-mediated H-bonds to the dC3pdG4 and dG2pdC3 phosphates, respectively (Figure 2a) [7]. The N173A mutant displays the most dramatic decrease in Z-DNA binding affinity, suggesting that it plays an important role in the Zα function [27,28]. Similarly, the K169A mutant also has a significantly lower Z-DNA binding affinity than a wild-type protein [27,28]. K170 forms direct H-bonds to the dG4pdC5 and dC5pdG6 phosphates (Figure 2a) [7]. The K170A mutant binds to Z-DNA with a lower affinity than wild-type protein, but better than the K169A and N173A mutants [27,28]. Interestingly, R174 and T191 bind to the furanose oxygens of dG6 and dG2, respectively (Figure 2a) [7]. However, the R174A and T191A mutations have little effect on the B-Z transition activities of hZα_ADAR1_ [28].

In addition to polar interactions, the aromatic ring of Y177 and the side-chains of P192 and P193 make the important van der Waals interactions with Z-DNA (Figure 2a) [7]. Interestingly, Y177 displays CH-π interaction with the C8 position of dG4 (Figure 2a) [7]. The Y177A mutant, which is unable to form both H-bonding and hydrophobic interactions, exhibits a significantly low Z-DNA binding affinity [27]. The Y177I, and Y177F mutants, which are capable only of hydrophobic interactions, could bind better to Z-DNA than Y177A, but still worse than wild-type protein [17,27,28]. Furthermore, the aromatic ring of W195, which forms a water-mediated H-bond to the dG2pdC3 phosphate, is almost perpendicular to Y177 and positioned in the center of the hydrophobic core (Figure 1c) [7]. The W195F mutant has 2-fold lower B-Z transition activity than wild type protein [28].

The crystal structural study reported that hZα_ADAR1_ is also able to bind to 6-bp Z-DNA duplexes with non-CG-repeat sequences, such as d(CACGTG)_2_, d(CGTACG)_2_, and d(CGGCCG)_2_ [34]. In these structures, N173, Y177, P192, P193, and W195 contribute to the recognition of Z-DNA-like CG-repeat DNA [34]. However, R174 and T191 did not show intermolecular interaction with Z-DNA in most structures [34]. Similarly, K169 and K170 are only in contact with Z-DNA within some structures of these complexes [34]. Thus, these four residues might play an important role in the sequence discrimination step for the B-Z transition of DNA.

The second Z-DNA binding domain of human ADAR1 (hZβ_ADAR1_) adopts a winged-HTH fold like hZα_ADAR1_, with the addition of a C-terminal α4 helix (see sequence in Figure 1a) [35]. Superposition of the hZβ_ADAR1_ with the hZα_ADAR1_ structure reveals that A327, A332, and I335 (corresponding to K169, R174, and Y177 in hZα_ADAR1_, respectively) did not perform H-bonding to the backbone of Z-DNA. Mutagenesis studies of these residues have shown that all three residues are important for Z-DNA-binding by Zα proteins [17,27,28]. This study suggested that hZβ_ADAR1_ is unable to interact with nucleic acids in a manner similar to that seen in the hZα_ADAR1_-Z-DNA complex [35]. Instead, this region participates in self-association protein–protein interactions [35].

### 2.2. mZα_DLM1_-Z-DNA Complex

The amino acid sequence of the Zα domain of murine DLM-1 (mZα_DLM1_) is ~35% identical to that of the hZα_ADAR1_ (Figure 1a). The overall structures of the complexes with Z-DNA are very similar to each other [29]. The core of the Zα-DNA interface in both proteins consists of three identical residues: N173, Y177, and W195 in hZα_ADAR1_ and N46, Y50, and W66 in mZα_DLM1_, respectively (Figure 2a,b) [7,29]. K43 and Q47 form direct or water-mediated H-bonds to three phosphates of the dC3pdG4pdC5pdG6 sequence like the corresponding K170 and R174 of hZα_ADAR1_ (Figure 2b) [29]. The structural differences between the two domains are found in the α1-β1 loop and the β-hairpin [29]. The β-hairpin of mZα_DLM1_ is two residues shorter than that of hZα_ADAR1_ (Figure 1a), indicating that the β-hairpin is apparently tolerant of greater sequence variability than the α3 helix without loss of function [29].

### 2.3. yabZα_E3L_-Z-DNA Complex

The Zα domain of the Yaba-like disease virus E3L (yabZα_E3L_) stabilizes the Z-DNA conformation in a manner similar to that of hZα_ADAR1_ and mZα_DLM1_ [30], although it shares only 26% sequence identity with hZα_ADAR1_ (Figure 1a). The crystal structural study revealed that two yabZα_E3L_ domains are found in the asymmetric unit, each bound to one strand of double-stranded DNA in the Z- conformation [30]. The intermolecular interaction of one asymmetric unit with Z-DNA is summarized in Figure 2c [30]. Three residues, N47, Y51, and W69 (corresponding to N173, Y177, and W195 in hZα_ADAR1_), play central roles in the interaction with Z-DNA, as with other members of the Zα family (Figure 2c). K43, K44, and Q48 also participate in DNA recognition via direct or water-mediated H-bonds to the phosphate backbone of Z-DNA (Figure 2c).

### 2.4. caZα_PKZ_-Z-DNA Complex

The Zα domain of PKZ from *Carassius auratus* (caZα_PKZ_), which shows limited identity with other ZBPs (28% for hZα_ADAR1_, 20% for hZα_DAI_, and 22% for yabZα_E3L_, respectively), is able to convert d(CG)-repeat DNA from B-DNA to Z-DNA [36,37]. The interaction between caZα_PKZ_ and Z-DNA is mediated by five residues in the α3 helix and four residues in the β-hairpin, similar to other Zα proteins (Figure 2d) [31]. Unlike the positively charged Lys or Arg in other ZBPs, the S35 in the α3 helix forms electrostatic interaction with the dC3pdG4 phosphate (Figure 2d) [31]. Interestingly, K56 of caZα_PKZ_ interacts not only with dC1pdG2 but also with dT0pdC1 (Figure 2d) [31], whereas a polar residue, like Ser or Thr at the corresponding position in other mammalian ZBPs, could not form these interactions (Figure 2). Generally, the B-Z transition activity by ZBPs was decreased when the ionic strength was increased. Surprisingly, the reduction of the B-Z transition rate is more severe in caZα_PKZ_ than in hZα_ADAR1_, suggesting that the effect of charge-charge interactions on B-to-Z transition activity plays a more critical role in the case of caZα_PKZ_ [31].

### 2.5. hZβ_DAI_-Z-DNA Complex

The second ZBD of human DAI (hZβ_DAI_) was also shown to bind Z-DNA based on its binding specificity for Z-DNA and its ability to convert B-DNA to Z-DNA [16]. Although hZβ_DAI_ also has α/β topology with three helices packed against three β-stands, like other ZBPs, hZβ_DAI_ has a 3_10_ helix at the N terminus of α3, instead of the long continuous α3 helix [32]. In the hZβ_DAI_-Z-DNA complex, protein-DNA interactions are mediated by the most conserved core residues, N141, Y145, and W162 (corresponding to N173, Y177, and W195) [32]. However, except for those core residues, other interactions with Z-DNA seem to be different for hZβ_DAI_ (Figure 2) [32]. For example, K138, located in the region between K169 and K170 of hZα_ADAR1_, forms an H-bond to the dC3pdG4 phosphate of one Z-DNA strand, whereas the two Lys residues of hZα_ADAR1_ contact the 4 phosphate groups of Z-DNA (Figure 2) [32]. Interestingly, K138 spans the length of the Z-DNA molecule and interacts with the dC5pdG6 phosphate on the opposite DNA strand (Figure 2e) [32]. An NMR study found that free hZβ_DAI_ has notable alterations in the α3 helix, the β-hairpin, and Y145 which are critical in Z-DNA recognition [38]. These results indicate that, unlike some other Zα domains, structural flexibility of hZβ_DAI_ is required for Z-DNA binding [38].

### 2.6. hZα_ADAR1_-Z-RNA Complex

ADAR1 edits dsRNA in vitro at significantly higher levels when dsRNA contains the purine-pyrimidine repeat sequence in dsRNA [39]. The hZα_ADAR1_ protein can bind to Z-RNA like Z-DNA [40]. It was first reported that the crystal structure of hZα_ADAR1_ complexed with 6-bp dsRNA, r(UCGCGCG)_2_ [33]. Interestingly, hZα_ADAR1_ exhibited significantly different binding modes when bound to Z-RNA versus Z-DNA (Figure 2). First, in the Z-RNA binding conformation, Y177 showed H-bonding interaction with the rG2prC3 phosphate and the O2’ of rG2, whereas it H-bonded with only the dG2pdC3 phosphate in the Z-DNA binding structure (Figure 2) [7,33]. Second, in the Z-DNA binding structure, R174 showed a direct, water-mediated H-bonding interactions with the dC5pdG6 phosphate and the O4’ of dG6, respectively (Figure 2a) [7]. However, when binding to Z-RNA, a water-mediated H-bond with the rC5prG6 phosphate as well as an H-bond with the E171 side-chain were formed (Figure 2f) [33]. Third, in the Z-RNA binding conformation, H159 exhibited a distinct orientation due to a water-mediated H-bonding interaction with the K169 side-chain compared to the Z-DNA binding conformation [33]. Fourth, in the Z-RNA binding conformation, T191 showed H-bonding interaction with only the rC3prG4 phosphate, whereas it formed H-bonds with both the phosphate and the O4’ of dC3 in the Z-DNA binding structure (Figure 2) [7,33].

## 3. Molecular Mechanism of B-Z Transition of 6-bp DNA Induced by ZBPs

### 3.1. B-Z Transition of a 6-bp CG-Repeat DNA by hZα_ADAR1_

**Figure 3:**
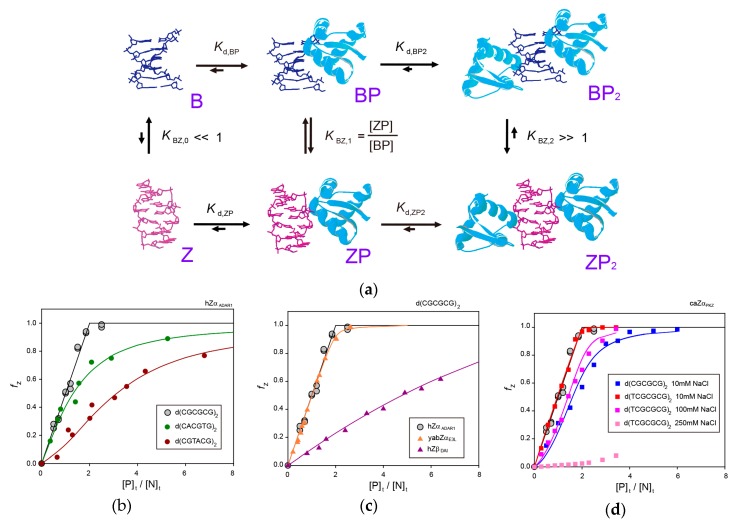
(**a**) Mechanism for the B-Z conformational transition of a 6-bpDNA by two ZBPs [41]. (**b**) Relative Z-DNA populations (*f*_Z_) of d(CGCGCG)2 (grey circle) [41], d(CACGTG)_2_ (dark green circle) [42], and d(CGTACG)_2_ (brown circle) [42] induced by hZα_ADAR1_ in NMR buffer (pH = 8.0) containing 100 mM NaCl as a function of [P]_t_/[N]_t_ ratio. (**c**) *f*_Z_ of d(CGCGCG)_2_ induced by hZα_ADAR1_ (grey circle) [41], yabZα_E3L_ (orange triangle) [43], and hZβ_DAI_ (purple triangle) [44] in NMR buffer (pH = 8.0) containing 100 mM NaCl as a function of [P]_t_/[N]_t_ ratio. (**d**) *f*_Z_ of d(CGCGCG)_2_ at 10 mM NaCl (blue square) and d(TCGCGCG)_2_ at 10 mM (red square), 100 mM (pink square), and 250 mM NaCl (light pink square) induced by caZα_PKZ_ [45].

NMR studies on the hZα_ADAR1_-Z-DNA interaction first proposed the B-Z transition mechanism of a 6-bp DNA, d(CGCGCG)_2_, by hZα_ADAR1_, in which the hZα_ADAR1_ plays two independent roles: (i) one molecule first binds to B-DNA and shifts the equilibrium from B-DNA to Z-DNA (BP to ZP, where B, Z, and P indicate B-DNA, Z-DNA, and protein); and (ii) the second molecule selectively binds to and stabilizes the Z-DNA conformation (Figure 3a) [41]. This study confirmed the existence of a one-to-one complex of Z-DNA and hZα_ADAR1_ (ZP) by gel filtration chromatography, NMR dynamics data, and diffusion coefficient values as functions of the [P]_t_/[N]_t_ molar ratio, where [P]_t_ and [N]_t_ are the total concentrations of the hZα_ADAR1_ and DNA, respectively [41]. It was found that the Z-DNA produced was half the total amount of the added hZα_ADAR1_ when the [P]_t_/[N]_t_ ratio was ≤2 (that is Z_t_ = 1/2[P]_t_), where Z_t_ is the total amount of the Z-DNA conformation (Figure 3b) [41]. To satisfy this relation, the BP and ZP complexes must exist as intermediate states with a correlation of [ZP] = [BP] (that is *K*_BZ,1_ = [ZP]/[BP] ≈1). Based on this correlation, the observed exchange rate constant (*k*_ex_) for the imino proton in the Z-DNA conformation could be expressed as a function of the [P]_t_/[N]_t_ ratio by the following Equation:

(1) $$k_{\mathrm{ex}}=k_{\mathrm{ex},\mathrm{ZP}2}+\frac{k_{\mathrm{ex},\mathrm{ZP}}-k_{\mathrm{ex},\mathrm{ZP}2}}{\left(1-\alpha \right)\chi }\left\{1-\sqrt{1-4\left(1-\alpha \right)\left(\frac{\chi }{2}-\frac{\chi^{2}}{4}\right)}\right\}$$

where *k*_ex,ZP_ and *k*_ex,ZP2_ are the exchange rate constants of the imino protons for the ZP and ZP_2_ complexes, respectively, χ is the [P]_t_/[N]_t_ ratio, and α (= *K*_d,ZP2_/*K*_d,BP_) is the ratio of the dissociation constants of BP and ZP_2_ complexes [41]. The *k*_ex_ dataset was fitted using Equation (1) to obtain the α value of 1.15 × 10^−2^ (Table 1) [41].

### 3.2. B-Z Transition of a 6-bp Non-CG-Repeat DNA by hZα_ADAR1_

The hZα_ADAR1_ protein can also convert the B-form of non-CG-repeat DNA, d(CACGTG)_2_ and d(CGTACG)_2_, to Z-form with lower activities compared to CG-repeat DNA, d(CGCGCG)_2_ (Figure 3b) [41,42]. Equation (1) could not be used to analyze the hydrogen exchange data of these DNA complexed with hZα_ADAR1_ because the non-CG-repeat DNA did not satisfy the relation, Z_t_ = 1/2[P]_t_. Instead, the observed *k*_ex_ value for the imino proton in the B-DNA (not Z-DNA) conformation could be expressed as a function of the relative Z-DNA population (*f*_Z_ = Z_t_/[N]_t_) by the following equation:

(2) $$k_{\mathrm{ex}}=k_{\mathrm{ex},B}+\frac{k_{\mathrm{ex},\mathrm{BP}}-k_{\mathrm{ex},B}}{2\left(1-\alpha \right)\left(1-f_{z}\right)}\left\{1+\left(K_{\mathrm{BZ},1}-1\right)f_{z}-\sqrt{{\left(1+\left(K_{\mathrm{BZ},1}-1\right)f_{z}\right)}^{2}-4K_{\mathrm{BZ},1}\left(1-\alpha \right)f_{z}\left(1-f_{z}\right)}\right\}$$

where *k*_ex,B_ and *k*_ex,BP_ are the exchange rate constants of the imino protons for free B-DNA and the BP complex, respectively [42]. The NMR dynamics studies found that hZα_ADAR1_ binds to non-CG-repeat DNA with weak binding affinity through the α3 helix as well as through the loop-β1-loop (151–158) and the α3-loop-β2 regions (178–191) [46]. Then, the B-form helix of non-CG-repeat DNA duplexes can be converted to a Z-conformation via these multiple intermolecular interactions with hZα_ADAR1_ proteins [46]. These studies explained how hZα_ADAR1_ exhibited the sequence preference of d(CGCGCG)_2_ >> d(CACGTG)_2_ > d(CGTACG)_2_ during the B-Z transition [42,46]. First, the P binds to the B, with a sequence preference of d(CGCGCG)_2_ >> d(CACGTG)_2_ > d(CGTACG)_2_ [42], even though the structural features of these three DNA duplexes complexed with hZα_ADAR1_ are very similar to each other [34]. Second, the BP of d(CGCGCG)_2_ and d(CACGTG)_2_ convert to ZP. In d(CGTACG)_2_, however, this process is less efficient as a way of discriminating d(TA)-containing DNA sequences from alternating pyrimidine-purine sequences [42]. Third, the ZP of d(CGCGCG)_2_ and d(CACGTG)_2_ binds to the P and forms the stable ZP_2_ complex with a sequence preference of d(CGCGCG)_2_ >> d(CACGTG)_2_, which acts as the third sequence discrimination step [42]. Taken together, it was suggested that hZα_ADAR1_ selectively recognizes the alternating d(CG)_n_ sequence and then converts it to a Z-form helix in long genomic DNA through its multiple sequence discrimination steps [42,46].

### 3.3. B-Z Transition of a 6-bp DNA by yabZα_E3L_

The yabZα_E3L_ could efficiently change the B-form helix of the d(CGCGCG)_2_ to left-handed Z-DNA like hZα_ADAR1_ (Figure 3c) [43]. In this study, because the B-Z transition activity of yabZα_E3L_ did not satisfy the relation, Z_t_ = 1/2[P]_t_, the observed *k*_ex_ value for the imino proton in the Z-DNA conformation could be expressed by the following equation instead of Equation (1):

(3) $$k_{\mathrm{ex}}=k_{\mathrm{ex},\mathrm{ZP}2}+\frac{k_{\mathrm{ex},\mathrm{ZP}}-k_{\mathrm{ex},\mathrm{ZP}2}}{2K_{\mathrm{Bz},1}\left(1-\alpha \right)f_{z}}\left\{1+\left(K_{\mathrm{BZ},1}-1\right)f_{z}-\sqrt{{\left(1+\left(K_{1,\mathrm{BZ}}-1\right)f_{z}\right)}^{2}-4K_{\mathrm{BZ},1}\left(1-\alpha \right)f_{z}\left(1-f_{z}\right)}\right\}$$

The *k*_ex_ dataset was fitted using Equation (3) to obtain the α value of 0.154 and the *K*_BZ,1_ value of 1.02 (Table 1) [43]. This *K*_BZ,1_ value means that yabZα_E3L_ and hZα_ADAR1_ have the same B-Z transition efficiency [43], which is consistent with their structural similarity in complexes with Z-DNA [7,30].

### 3.4. B-Z Transition of a 6-bp DNA by hZβ_DAI_

NMR studies have revealed that hZβ_DAI_ had significantly lower B-Z transition activity than hZα_ADAR1_ and yabZα_E3L_ (Figure 3c) [44]. In addition, the imino proton and ^31^P-NMR spectra of d(CGCGCG)_2_ complexed with hZβ_DAI_ are completely different from those of the d(CGCGCG)_2_-hZα_ADAR1_ complex [44]. These indicate that the base pair geometry and backbone conformation of the hZβ_DAI_-induced Z-DNA helix are significantly different from those of the Z-DNA-hZα_ADAR1_ complex, similar to their crystal structures [7,32]. The hydrogen exchange study of the d(CGCGCG)_2_-hZβ_DAI_ complex found that the exchange rates of imino protons in B-DNA as well as Z-DNA conformations are not affected by complex formation [A4]. In addition, diffusion optimized spectroscopy experiments confirmed that the Z-form of d(CGCGCG)_2_ complexed with hZβ_DAI_ exhibited one major complex state (perhaps ZP_2_), even at various [P]_t_/[N]_t_ [44]. Based on these results, they proposed the distinct B-Z transition mechanism where two molecules of hZβ_DAI_ initially bind directly to the B-form DNA and form the BP_2_ complex; subsequently, there is a conformational change from BP_2_ to ZP_2_. (Figure 3a) [44].

### 3.5. B-Z Transition of a 6-bp DNA by caZα_PKZ_

The caZα_PKZ_ domain can convert the dsDNA, d(CGCGCG)_2_ to Z-DNA with lower activity rates than hZα_ADAR1_ and yabZα_E3L_ (Figure 3d) [45]. Instead, caZα_PKZ_ exhibits full B-Z transition activity when binding to d(TCGCGCG)_2_ (Figure 3d) [45]. This indicates that the H-bonding interaction of K56 with the dT0pdC1 phosphate plays an important role in the B-Z transition of DNA by caZα_PKZ_ [45]. In this study, instead of the *k*_ex_ value, the ^1^H and ^15^N chemical shift changes (Δδ_obs_) of amide protons of caZα_PKZ_ and relative Z-DNA population (*f*_Z_) were determined as the functions of [N]_t_ and [P]_t_ expressed by the following functions, respectively:

(4) $${\Delta \delta }_{\mathrm{obs}}=\frac{\left[\mathrm{BP}\right]}{{\left[P\right]}_{t}}{\Delta \delta }_{B}+\frac{\left[\mathrm{ZP}\right]+2\left[{\mathrm{ZP}}_{2}\right]}{{\left[P\right]}_{t}}{\Delta \delta }_{Z}$$

(5) $$f_{Z}=\frac{\left[\mathrm{ZP}\right]+\left[{\mathrm{ZP}}_{2}\right]}{{\left[P\right]}_{t}}$$

where Δδ_B_ and Δδ_Z_ are the ^1^H and ^15^N chemical shift differences of the B-DNA- and Z-DNA-bound forms relative to free form, respectively, [BP], [ZP], and [ZP_2_] are the concentration of the BP, ZP, and ZP_2_ complex states, respectively, which are described as:

(6) $$\left[\mathrm{BP}\right]={\left[N\right]}_{t}\frac{K_{d,\mathrm{ZP}2}\left[P\right]}{K_{d,\mathrm{BP}}K_{d,\mathrm{ZP}2}+\left(1+K_{\mathrm{BZ},1}\right)K_{d,\mathrm{ZP}2}\left[P\right]+K_{\mathrm{BZ},1}{\left[P\right]}^{2}}$$

(7) $$\left[\mathrm{ZP}\right]={\left[N\right]}_{t}\frac{K_{\mathrm{BZ},1}K_{d,\mathrm{ZP}2}\left[P\right]}{K_{d,\mathrm{BP}}K_{d,\mathrm{ZP}2}+\left(1+K_{\mathrm{BZ},1}\right)K_{d,\mathrm{ZP}2}\left[P\right]+K_{\mathrm{BZ},1}{\left[P\right]}^{2}}$$

(8) $$\left[{\mathrm{ZP}}_{2}\right]={\left[N\right]}_{t}\frac{K_{\mathrm{BZ},1}{\left[P\right]}^{2}}{K_{d,\mathrm{BP}}K_{d,\mathrm{ZP}2}+\left(1+K_{\mathrm{BZ},1}\right)K_{d,\mathrm{ZP}2}\left[P\right]+K_{\mathrm{BZ},1}{\left[P\right]}^{2}}$$

and [P] is the concentration of free caZα_PKZ_, solvable via the following cubic equation [45]:

(9) $${\left[P\right]}^{3}+a{\left[P\right]}^{2}+b\left[P\right]+c=0$$

(10) $$a=2{\left[N\right]}_{t}-{\left[P\right]}_{t}+\left(1+\frac{1}{K_{\mathrm{BZ},1}}\right)K_{d,\mathrm{ZP}2}$$

(11) $$b=\left(1+\frac{1}{K_{\mathrm{BZ},1}}\right)K_{d,\mathrm{ZP}2}\left({\left[N\right]}_{t}-{\left[P\right]}_{t}\right)+\frac{K_{d,\mathrm{BP}}K_{d,\mathrm{ZP}2}}{K_{\mathrm{BZ},1}}$$

(12) $$c=-\frac{K_{d,\mathrm{BP}}K_{d,\mathrm{ZP}2}}{K_{\mathrm{BZ},1}}{\left[P\right]}_{t}$$

The closed-form solution of Equation (9) has been expressed by [45]:

(13) $$\left[P\right]=-\frac{a}{3}+\frac{2}{3}\sqrt{a^{2}-3b}\cos\frac{\theta }{3}$$

where

(14) $$\theta =\arccos\left(\frac{-2a^{3}+9ab-27c}{2\sqrt{{\left(a^{2}-3b\right)}^{2}}}\right)$$

In order to obtain accurate *K*_d_ values, all ^1^H and ^15^N titration curves and the *f*_Z_ data were globally fitted with Equation (4) and Equation (5), respectively. This approach successfully provides two binding constants, *K*_d,BP_ and *K*_d,ZP2_, not the relative ratio (α) of these two constants as in previous studies.

At 10 mM NaCl, the global fitting gave a *K*_d,BP_ and a *K*_d,ZP2_ of 28 and 350 nM, respectively, and a *K*_BZ,1_ of 0.87 (Table 2) [45]. As [NaCl] was increased, the *K*_d,BP_ and *K*_d,ZP2_ values became increased, but the *K*_BZ,1_ value became smaller (Table 2) [45,47]. The NMR dynamics studies found that increasing the ionic strength interferes more with the association of ZP with caZα_PKZ_ via intermolecular electrostatic interactions rather than the dissociation of ZP_2_ [45]. In addition, the global fitting method using Equation (4) also provides the ^1^H and ^15^N chemical shift differences between the free and the bound forms for both B-DNA (Δδ_B_) and Z-DNA binding (Δδ_Z_). At higher concentrations of NaCl, the B-DNA-bound state exhibited completely different results than at 10 mM NaCl, whereas the Z-DNA binding conformation was not affected by the change of ionic strength [45,47]. These results meant that the B-DNA binding state of caZα_PKZ_ exhibited distinct structural features under high and low salt conditions which might be related to reduced B-Z transition activity at higher [NaCl]. Taken together, these studies suggest that the intermediate complex formed by caZα_PKZ_ and B-DNA can be used as a molecular ruler to measure the degree to which DNA transitions to the Z isoform [45,47].

## 4. Conclusions

Z-DNA is induced by various Z-DNA binding proteins (ZBPs) that play important roles in RNA editing, innate immune response, and viral infection. We summarized the structural and dynamics data of various ZBPs complexed with Z-DNA to understand the mechanisms by which ZBPs selectively recognize d(CG)-repeat DNA sequences in genomic DNA and efficiently convert it to left handed Z-DNA to achieve their biological function. The contact of ZBPs with Z-DNA strands mediated through a single continuous recognition surface consists of an α3 helix and a β-hairpin. In the ZBP-Z-DNA complexes, three conserved identical residues of ZBPs (N173, Y177, and W195 in hZα_ADAR1_) play a central role in interactions with Z-DNA. ZBPs convert a 6-bp DNA to a Z-form helix via a B-Z transition mechanism in which the ZBP first binds to B-DNA and then shifts the equilibrium from B-DNA to Z-DNA, a conformation that is then selectively stabilized by the additional binding of a second ZBP molecule. During B-Z transition, ZBPs selectively recognize the alternating d(CG)_n_ sequence and then convert it to a Z-form helix in long genomic DNA through its multiple sequence discrimination steps. In addition, the intermediate complex formed by ZBPs and B-DNA, which is modulated by varying conditions, determines the degree of B-Z transition.

## Figures and Tables

**Table 1:** Equilibrium constants for the ZBP-induced B-Z transition.

ZBP	DNA	α ^1^	*K*_BZ,1_	*K*_d,BP_ (μM)	*K*_d,ZP2_ (μM)	Rerefences
hZα_ADAR1_	d(CGCGCG)_2_	1.15 × 10^−2^	~1	<0.1	<0.1	[41]
hZα_ADAR1_	d(CACGTG)_2_	1.42	0.4	260 ± 87	180 ± 62	[42]
hZα_ADAR1_	d(CGTACG)_2_	13.9	6.3	400 ± 144	29 ± 11	[42]
yabZα_E3L_	d(CGCGCG)_2_	0.154	1.02	n.d. ^2^	n.d. ^2^	[43]

^1^ α = *K*_d,ZP2_/*K*_d,BP_; ^2^ n.d.: not determined.

**Table 2:** Equilibrium constants for the caZα_PKZ_-induced B-Z transition.

ZBP	DNA	pH	[NaCl]	*K*_BZ,1_	*K*_d,BP_ (μM)	*K*_d,ZP2_ (μM)	Rerefences
caZα_PKZ_	d(TCGCGCG)_2_	6.0	10 mM	0.87 ± 0.03	0.028 ± 0.017	0.345 ± 0.079	[45]
caZα_PKZ_	d(TCGCGCG)_2_	6.0	100 mM	0.19 ± 0.02	16.4 ± 0.8	8.76 ± 0.67	[45]
caZα_PKZ_	d(TCGCGCG)_2_	6.0	250 mM	~0.01	64.1 ± 8.3	9.57 ± 0.85	[47]
caZα_PKZ_	d(TCGCGCG)_2_	8.0	10 mM	1.18 ± 0.03	0.157 ± 0.021	0.129 ± 0.074	[45]
caZα_PKZ_	d(TCGCGCG)_2_	8.0	100 mM	0.18 ± 0.02	5.41 ± 0.66	2.41 ± 0.37	[45]
caZα_PKZ_	d(CGCGCG)_2_	8.0	10 mM	0.11 ± 0.05	5.18 ± 2.43	1.79 ± 0.95	[45]
